# Prescribing patterns of coronary artery aneurysm in Taiwan

**DOI:** 10.1186/s12872-019-1172-6

**Published:** 2019-08-05

**Authors:** Chun-Hui Lu, Chih-Wun Fang, Hao-Ming Chen, Yi-Ping Fang, Chein-Tang Fang, Yaw-Bin Huang, Chung-Yu Chen, Kuang-Ming Liao, Shu-Chuan Jennifer Yeh

**Affiliations:** 1 Division of Pharmacy, Zuoying Branch of Kaohsiung Armed Forces General Hospital, Kaohsiung, Taiwan; 2 Division of Health Technology Assessment, Center for Drug Evaluation, Taipei, Taiwan; 3 School of Pharmacy, Kaohsiung Medical University, 100, Shiquan 1st Rd., Sanmin Dist.,, Kaohsiung City, 80708 Taiwan, Republic of China; 4 Department of Pharmacy, Kaohsiung Medical University Hospital, Kaohsiung, Taiwan; 5 Department of Medical Research, Kaohsiung Medical University Hospital, Kaohsiung, Taiwan; 6 Department of Internal Medicine, Chi Mei Medical Center, Chiali, No.606, Jialixing, Jiali Dist., Tainan City, 72263 Taiwan, Republic of China; 7 Department of Business Management, National Sun Yat-sen University, Kaohsiung, Taiwan

**Keywords:** Comorbidities, Coronary artery aneurysm, Prescribing pattern

## Abstract

**Background:**

Coronary artery aneurysm (CAA) is a rare disease, and there are limited data on prescribing patterns for CAA. The aim of our study was to investigate prescribing patterns for CAA in Taiwan via the National Health Insurance Research Database (NHIRD).

**Methods:**

We included all CAA patients in Taiwan from 2005 to 2011. Data from 1 year before and after the CAA diagnosis were used to analyze examinations, comorbidities and prescribing patterns.

**Results:**

A total of 1397 patients diagnosed with CAA were enrolled in our study. Most pediatric patients with CAA were diagnosed with Kawasaki disease (95.7%). In pediatric CAA patients, the utilization rates of aspirin and gamma globulins were 82.9 and 53.6%, respectively, after CAA diagnosis. Among the antithrombotic agents, aspirin was used most commonly, followed by dipyridamole (16.9%), heparin (5.8%) and warfarin (4.6%). In adult CAA patients, common comorbidities included hypertension (63.4%), hyperlipidemia (39.6%), and diabetes mellitus (26.1%). Coronary atherosclerosis was identified in 72.5% of adult patients after CAA diagnosis. Antithrombotic agents, particularly aspirin, clopidogrel and heparin, were prescribed more frequently after CAA diagnosis. Among the prescribed medications, aspirin (75.8%), β-blockers (48.3%), statins (47.6%), metformin (14.4%), sulfonylureas (14.4%) and isosorbide mononitrate (32.9%) were frequently observed in each category.

**Conclusions:**

Kawasaki disease was the main cause of CAA in pediatric patients, and coronary artery disease was the most common comorbidity in adult CAA patients. The most commonly used antithrombic agent after CAA diagnosis was aspirin in both adult and pediatric patients.

## Background

Coronary artery aneurysm (CAA) is a rare disease that can manifest at any age; CAA was first described by Morgagni in 1761. [1] Typically, CAA is identified accidentally during diagnostic coronary angiography when patients have ischemic syndromes. CAA is defined as a dilatation of the blood vessel in which the luminal diameter exceeds more than 1.5-fold the size of the normal adjacent segments or the largest coronary vessel based on the Coronary Artery Surgery Study (CASS) registry [2].

The incidence of CAA varies widely, from 0.3 to 5.3% based on the literature [3–10]. Even though the prognosis of patients with CAA is generally good, CAA can cause ischemic syndrome with the presentation of angina, myocardial infarction or even sudden death. [11–13] CAA is primarily caused by atherosclerosis and Kawasaki disease in adults and children, respectively [14–16]. Other causative factors include connective tissue disorders, [17] congenital anomalies, infection, specific drug reactions, trauma or iatrogenic interventions [18]. Currently, there are no consensus guidelines for the management of CAA, except for the population with Kawasaki disease [19]. CAA treatment options include medical, surgical and percutaneous interventions. Medical therapy with antiplatelet or anticoagulant drugs seems to be beneficial for preventing thrombus formation and distal embolization in patients with CAA [20, 21]. Some authors have indicated that statins may be beneficial in such a population based on the high ratio of atherosclerotic disease [22].

Antiplatelet and anticoagulant therapy may be necessary if thrombosis or embolism is a concern [21]. Until now, there have been limited data on prescribing patterns for CAA, and the aim of our study was to investigate the prescribing patterns for CAA in Taiwan via the National Health Insurance Research Database (NHIRD).

## Methods

### Data source

We performed a population-based retrospective study using data from the NHIRD in Taiwan. The National Health Insurance program was established in 1995, and more than 99.9% of Taiwan’s population was enrolled as of 2014. By applying to the National Health Insurance Research Institutes for the specific subject datasets of CAA, we obtained data for the whole CAA population in Taiwan and permission to use it to investigate the epidemiology and prescribing patterns for CAA.

All medical records, including outpatient care, inpatient care, emergency care and prescriptions, were utilized for the analysis in the current study.

### Population

To identify patients diagnosed with CAA, we use the International Classification of Diseases, Ninth Revision, Clinical Modification (ICD-9-CM) code 414.11, and we selected patients from 2005 to 2011. Data from 1 year before and after the CAA diagnosis was used to analyze examinations, comorbidities and prescribing patterns. Patients who had 2 outpatient diagnoses or 1 inpatient diagnosis between January 1, 2005, and December 31, 2011, were enrolled. The index date was defined as the date of the first diagnosis of CAA. As CAA was identified with the assistance of imaging by coronary angiography, echocardiography (echo), computed tomography (CT), magnetic resonance imaging (MRI) or during cardiac catheterization, patients with CAA received one of the above examinations as confirmation. In our study, we defined patients who received one of the examinations, including coronary angiography, echo, CT, MRI, and cardiac catheterization, within 90 days before or after the index date as having CAA.

For evaluation of concomitant disease, patients were divided into two groups, pediatric and adult populations, considering the difference in the nature of the disease. In the current study, we defined the pediatric population as patients younger than 20 years of age and the adult population as patients older than 20 years of age.

This study was approved by the Institutional Review Board of the Kaohsiung Medical University Hospital (KMUHIRB-EXEMPT-20130199) and Kaohsiung Armed Forces General Hospital (KAFGH 106–039). Informed consent was waived because patient data were deidentified by scrambling the identification codes.

### Prescribing patterns

We recorded the medication use in the population and identified drugs administered 1 year before and after the index date by the Anatomical Therapeutic Chemical (ATC) Classification System. To investigate the usage of antithrombotic agents, prescriptions of common antiplatelets and anticoagulants used before or after the diagnosis of CAA were recorded and presented as numbers and proportions (%). In the analysis, antithrombotic agents included aspirin, clopidogrel, dipyridamole, ticlopidine, tirofiban, cilostazol, warfarin, heparin and enoxaparin. Oral drugs for cardiovascular disease, including antithrombotic agents, antihypertensive agents, antidyslipidemic agents, antidiabetic agents and nitrates, were also investigated.

### Statistical analysis

Age is presented as years (means ± standard deviations) and is divided into 3 groups: younger than 20 years, 20–64 years and older than 65 years. The detailed age distribution of the pediatric and adult populations is shown as a bar chart. Gender and other demographic information, including area, urbanization, and insurance premiums, is presented as numbers and proportions (%). Data processing and statistical analyses were performed with the use of SAS version 9.4 (SAS Inc., Cary, NC).

## Results

### Study population

There were 1493 patients with at least 2 CAA diagnoses at the outpatient department or 1 CAA diagnosis at the inpatient department from 2005 to 2011. After excluding 96 patients without any record of related examinations, a total of 1397 patients with CAA during 2005 to 2011 were identified and enrolled in our study. Patient characteristics are shown in Table 1. Among the 1397 CAA patients, the mean age was 37.76 ± 31.45 years. There were 586 (41.9%) pediatric patients aged less than 20 years, 430 (30.8%) adult patients aged 20 to 65 years and 381 (27.3%) adult patients aged 65 years and older.

**Table 1:** Baseline characteristics of the CAA population in Taiwan

Variable (*N* = 1397)	Number (%)
Age, year (mean ± SD)	37.76 ± 31.45
Age group (year)	
< 20	586 (41.9)
20–64	430 (30.8)
≥ 65	381 (27.3)
Gender	
Male	957 (68.5)
Female	440 (31.5)
Area	
North	546 (39.1)
Central	518 (37.1)
South	307 (22.0)
East	26 (1.9)
Urbanization	
Urbanized	1019 (72.9)
Rural	378 (27.1)
Intervention	
PCI	102 (7.3)
CABG	5 (0.4)
Monthly insurance premiums (NTD)	
< 20,000	1030 (73.7)
20,000–39,999	273 (19.5)
≥ 40,000	94 (6.7)

*NTD* New Taiwan Dollars, *PCI* percutaneous coronary intervention, *CABG* Coronary artery bypass grafting

Table 2 shows the medical procedures of the CAA population. Most adult patients (75.4%) underwent cardiac catheterization, and as high as 98% of pediatric patients received echo.

**Table 2:** Procedures received in the CAA population

Procedure	Age < 20 years (*n* = 592)	Age ≥ 20 years (*n* = 901)	All population (*n* = 1493)
Single item			
Angio	42 (7.1%)	405 (45.0%)	447 (29.9%)
Cath	59 (10.0%)	679 (75.4%)	738 (49.4%)
Echo	580 (98.0%)	578 (64.2%)	1158 (77.6%)
CT	42 (7.1%)	210 (23.3%)	252 (16.9%)
MRI	7 (1.2%)	48 (5.3%)	55 (3.7%)
Multiple items			
Angio or Cath	60 (10.1%)	680 (75.5%)	740 (49.6%)
Angio or Cath or Echo	586 (99.0%)	779 (86.5%)	1365 (91.4%)
Angio or Cath or Echo or CT or MRI	586 (99.0%)	811 (90.0%)	1397 (93.6%)^a^

^a^ Final population included in the epidemiological study

*Angio* coronary angiography, *Cath* cardiac catheterization, *Echo* echocardiography, *CT* computed tomography, *MRI* magnetic resonance imaging

### Comorbidities

Regarding the comorbidities of patients with CAA, we analyzed the 10 most common concomitant diseases based on different age groups (i.e., pediatric versus adult populations) and time frames (i.e., within 1 year before and after CAA diagnosis) to observe the disease status over time (Table 3). In the pediatric population, common coexisting diseases included acute upper respiratory infections (e.g., sinusitis, bronchitis and pharyngitis), acute febrile mucocutaneous lymph node syndrome (i.e., Kawasaki disease), noninfectious gastroenteritis and colitis and skin-related disorders. Notably, Kawasaki disease increased very quickly to a high level of frequency after the diagnosis of CAA; 95.7% of the pediatric population was diagnosed with Kawasaki disease.

**Table 3:** Comorbidities of CAA patients before and after CAA diagnosis

Comorbidity	1 year before index date N (%)	1 year after index date N (%)
Pediatric population, aged < 20 years (*N* = 586)		
Kawasaki disease	243 (41.5)	561 (95.7)
Acute respiratory infections	540 (92.2)	556 (94.9)
Noninfectious gastroenteritis and colitis	163 (27.8)	160 (27.3)
Pneumonia	82 (14.0)	122 (20.8)
Allergic rhinitis	66 (11.3)	97 (16.6)
Contact dermatitis	98 (16.7)	80 (13.7)
Urinary tract infection	70 (11.9)	66 (11.3)
Atopic dermatitis	63 (10.8)	55 (9.4)
Influenza	35 (6.0)	47 (8.0)
Infectious colitis, enteritis and gastroenteritis	37 (6.3)	38 (6.5)
Adult population, age ≥ 20 years (*N* = 811)		
Coronary artery disease	533 (65.7)	704 (86.8)
Coronary atherosclerosis	274 (33.8)	588 (72.5)
Hypertension	514 (63.4)	561 (69.2)
Dyslipidemia	321 (39.6)	399 (49.2)
Diabetes mellitus	212 (26.1)	255 (31.4)
Angina	153 (18.9)	238 (29.3)
Heart failure	107 (13.2)	171 (21.1)
Arrhythmia	131 (16.2)	159 (19.6)
Myocardial infarction	84 (10.4)	152 (18.7)
Chronic obstructive pulmonary disease	127 (15.7)	139 (17.1)

In the adult population, most of the comorbidities before CAA diagnosis were chronic diseases. Cardiovascular diseases, such as coronary artery disease (65.7%), hypertension (63.4%), dyslipidemia (39.6%) and diabetes (26.1%), were very common in this population. The rates of several coexisting diseases were higher after the index date than before, among which coronary atherosclerosis predominated, with an increase from 33.8 to 72.5% after CAA diagnosis.

### Prescribing patterns

The prescribing patterns in the CAA population were divided into 2 categories, the utilization of antithrombotic agents (Table 4) and long-term medications for cardiovascular diseases in adults (Table 5).

**Table 4:** Antithrombotic agents used in the CAA population before and after CAA diagnosis

	Before index date	After index date		
	1 year	3 month	6 month	1 year
	N (%)	N (%)	N (%)	N (%)
Pediatric population (*N* = 586)				
Aspirin	182 (31.1)	479 (81.7)	482 (82.3)	486 (82.9)
Clopidogrel	1 (0.2)	4 (0.7)	4 (0.7)	4 (0.7)
Dipyridamole	43 (7.3)	95 (16.2)	97 (16.6)	99 (16.9)
Warfarin	7 (1.2)	25 (4.3)	26 (4.4)	27 (4.6)
Heparin	5 (0.9)	31 (5.3)	33 (5.6)	34 (5.8)
Adult population (*N* = 811)				
Aspirin	575 (70.9)	667 (82.2)	678 (83.6)	689 (85.0)
Clopidogrel	256 (31.6)	510 (62.9)	514 (63.4)	523 (64.5)
Dipyridamole	127 (15.7)	81 (10.0)	94 (11.6)	108 (13.3)
Ticlopidine	32 (3.9)	26 (3.2)	31 (3.8)	36 (4.4)
Tirofiban	22 (2.7)	28 (3.5)	28 (3.5)	29 (3.6)
Cilostazol	20 (2.5)	26 (3.2)	29 (3.6)	41 (5.1)
Warfarin	60 (7.4)	126 (15.5)	132 (16.3)	141 (17.4)
Heparin	164 (20.2)	551 (67.9)	562 (69.3)	577 (71.1)
Enoxaparin	60 (7.4)	131 (16.2)	140 (17.3)	147 (18.1)

**Table 5:** Medication used in the CAA population before and after CAA diagnosis

	Before index date	After index date		
	1 year	3 month	6 month	1 year
N = 811	N (%)	N (%)	N (%)	N (%)
Antithrombotic agent	555 (68.4)	680 (83.8)	700 (86.3)	722 (89.0)
Aspirin	477 (58.8)	568 (70.0)	587 (72.4)	615 (75.8)
Clopidogrel	163 (20.1)	329 (40.6)	338 (41.7)	351 (43.3)
Dipyridamole	34 (4.2)	12 (1.5)	22 (2.7)	31 (3.8)
Cilostazol	13 (1.6)	18 (2.2)	20 (2.5)	29 (3.6)
Ticlopidine	12 (1.5)	10 (1.2)	16 (2.0)	20 (2.5)
Warfarin	33 (4.1)	76 (9.4)	97 (12.0)	109 (13.4)
Antihypertensive agent	599 (73.9)	613 (75.6)	675 (83.2)	703 (86.7)
ACEI	161 (19.9)	144 (17.8)	163 (20.1)	181 (22.3)
ARBs	234 (28.9)	229 (28.2)	261 (32.2)	300 (37.0)
CCBs	348 (42.9)	283 (34.9)	324 (40.0)	371 (45.7)
β-blockers	289 (35.6)	197 (24.3)	314 (38.7)	392 (48.3)
Diuretics	171 (21.1)	181 (22.3)	210 (25.9)	237 (29.2)
α-blockers	116 (14.3)	76 (9.4)	105 (12.9)	129 (15.9)
Antidyslipidemic agents	291 (35.9)	321 (39.6)	370 (45.6)	410 (50.6)
Statins	257 (31.7)	300 (37.0)	349 (43.0)	386 (47.6)
Fibrates	53 (6.5)	24 (3.0)	31 (3.8)	50 (6.2)
Others	11 (1.4)	13 (1.6)	14 (1.7)	18 (2.2)
Antidiabetic agents	177 (21.8)	144 (17.8)	170 (21.0)	187 (23.1)
Metformin	114 (14.1)	79 (9.7)	96 (11.8)	117 (14.4)
Sulfonylureas	132 (16.3)	98 (12.1)	107 (13.2)	117 (14.4)
Thiazolidinedione (TZD)	43 (5.3)	23 (2.8)	31 (3.8)	33 (4.1)
DDP-4 inhibitors	10 (1.2)	10 (1.2)	16 (2.0)	23 (2.8)
Others	45 (5.5)	28 (3.5)	38 (4.7)	49 (6.0)
Nitrates	265 (32.7)	290 (35.8)	350 (43.2)	388 (47.8)
Nitroglycerin	43 (5.3)	11 (1.4)	27 (3.3)	74 (9.1)
Isosorbide dinitrate	57 (7.0)	58 (7.2)	75 (9.2)	86 (10.6)
Isosorbide mononitrate	184 (22.7)	216 (26.6)	246 (30.3)	267 (32.9)
Nicorandil	41 (5.1)	24 (3.0)	44 (5.4)	69 (8.5)

*ACEI* angiotensin-converting-enzyme inhibitor, *ARB* angiotensin receptor blocker, *CCB* calcium channel blocker, *TZD* thiazolidinedione, *DDP-4 inhibitor* dipeptidyl peptidase-4 inhibitor

**Fig. 1:**
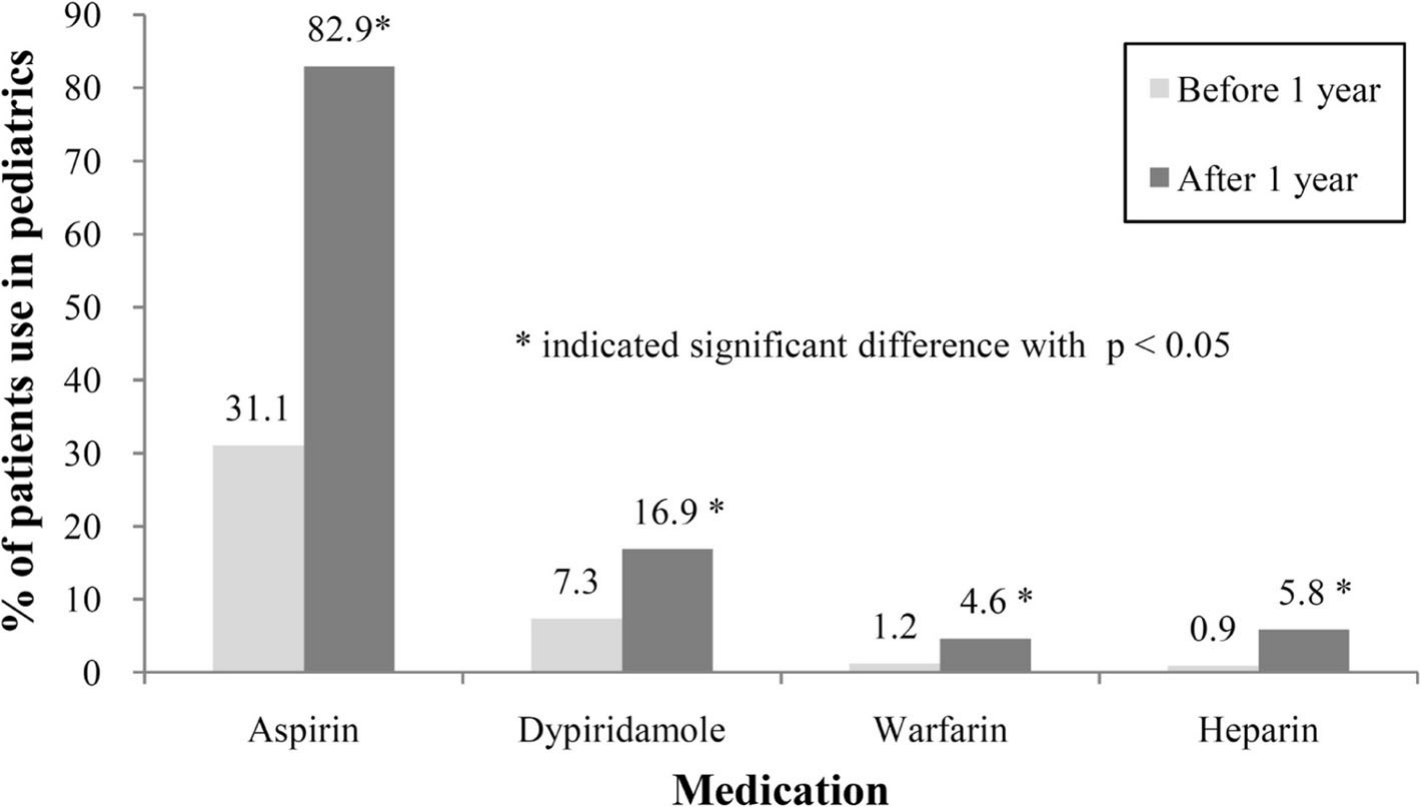
Antithrombotic agents used in pediatric population

In the pediatric population, the usage of aspirin (82.9%) and gamma globulins (53.6%) increased after CAA diagnosis, which reflected the need to attenuate inflammation of the vessels and prevent thrombosis in patients with Kawasaki disease. For antithrombotic agents, the most frequently used antiplatelet agent in the pediatric population was aspirin, followed by dipyridamole. A small proportion of pediatric patients had been prescribed warfarin and heparin. The prescribing rates of antithrombotic agents increased after CAA diagnosis in the pediatric population (Fig. 1).

**Fig. 2:**
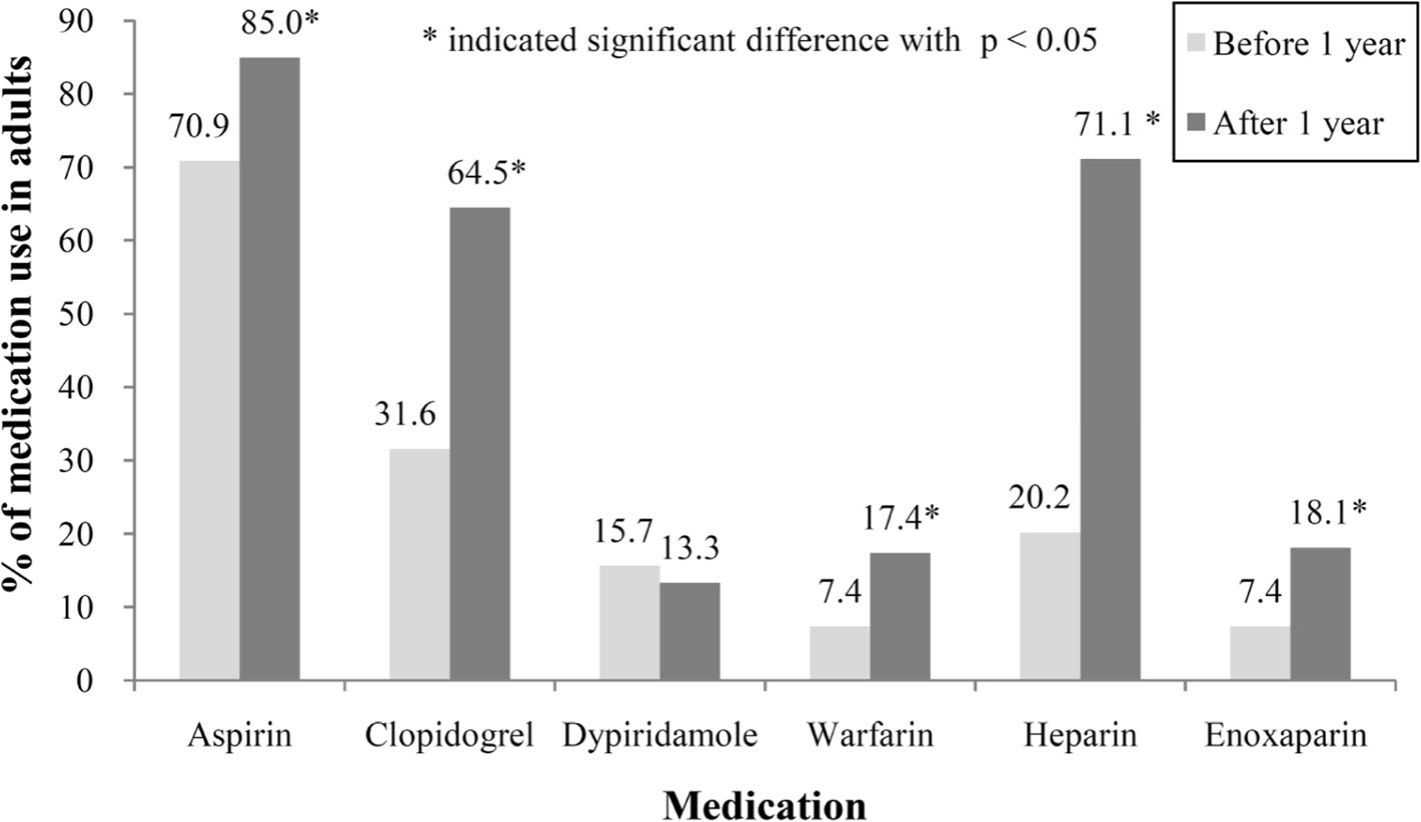
Antithrombotic agents used in the adult population

In the adult population, which differed from the pediatric population, antiplatelets (aspirin, clopidogrel) and nitrates (nitroglycerin, isosorbide 5-mononitrate) were frequently prescribed before CAA diagnosis in adults owing to underlying chronic diseases. Furthermore, aspirin, clopidogrel and heparin usage increased both in frequency and average amount after CAA diagnosis (Fig. 2).

Among the antiplatelet agents, the prescribing rate of aspirin was the highest, followed by clopidogrel, dipyridamole and other drugs. For anticoagulants, heparin was the most often prescribed; as many as 71.1% of adult patients had taken heparin. Nearly all antithrombotic agents, except for dipyridamole, had higher prescribing rates after CAA diagnosis than before (Table 4).

Considering the high proportion of cardiovascular disease in adults, we evaluated related oral drugs in long-term use. Table 5 shows the prescribing rates of each class of medication. As a whole, every kind of medication tended to increase after CAA diagnosis, except antidiabetic agents, which slightly decreased (Table 5). The most frequently prescribed drugs among each class were aspirin, β-blockers, statins, sulfonylureas (equal to metformin 1 year after index date) and isosorbide mononitrate.

## Discussion

This study included a total of 1397 patients with CAA during 2005–2011. The mean age of our population was 37.76 ± 31.45 years. Notably, the age distribution ranged widely from 0 to 91 years in our study, and the pediatric, adult and elderly populations accounted for 41.9, 30.8 and 27.3% of the total, respectively. In contrast with angiographic series in the literature, we had access to the data of pediatric patients with CAA instead of only adults. The mean age of the pediatric population was 3.16 ± 3.66 years, and approximately 69.3% of the pediatric patients were male.

### Comorbidities in pediatric patients

Most pediatric patients were diagnosed with Kawasaki disease (95.7%), especially infants 1 year of age and below (23.2%), and were under 5 years of age, which was in accordance with epidemiological reports in Taiwan [23–25].

Kawasaki disease is a systemic vasculitis that commonly involves the coronary arteries and is considered the main cause of nonatherosclerotic CAA in young children [20, 22]. From our results, the disease burden of Kawasaki disease in Taiwan and its associated risk for the development of CAA cannot be overlooked. For young patients presenting acute coronary syndrome, such as angina and myocardial infarction, the development of CAA should be considered and patients should receive treatment as soon as possible.

### Comorbidities in adult patients

The mean age of the adult population with CAA was 62.9 ± 14.1 years, and the majority of patients were male (67.9%). Prevalent coexisting diseases included hypertension (63.4%), hyperlipidemia (39.6%), diabetes mellitus (26.1%) and other chronic diseases. A small proportion of CAA patients had aortic dissection and aortic aneurysm. Most of the comorbidities were diagnosed more frequently after identification of CAA. Remarkably, coronary atherosclerosis was found in 72.5% of patients after CAA diagnosis.

Atherosclerosis remains the principal cause of CAA in the adult population, even though the exact etiology is still unknown [22, 26]. Our results showed a high proportion of coronary atherosclerosis (72.5%) in adults, which was consistent with the literature. With respect to cardiovascular diseases, our patients had a higher incidence of hypertension and diabetes mellitus but a lower incidence of hyperlipidemia than those in other reports in general. The variation may be due to the relatively older age of our population. Hypertension, hyperlipidemia and diabetes mellitus are risk factors known to be associated with atherosclerosis; however, the relationship between risk factors of hypertension, hyperlipidemia, diabetes mellitus and CAA needs to be further investigated.

Previous myocardial infarction and angina were reported in 10.4 and 18.9% of our population, respectively; these proportions were lower than those in some previous studies [21, 27, 28]. It has been suggested that patients with CAA commonly present with ischemic heart disease such as angina pectoris and myocardial infarction. Nevertheless, it was difficult to differentiate whether these symptoms were manifestations of aneurysm or coronary artery disease [22].

### Prescribing patterns in pediatric patients

In the current study, we found an increased utilization of aspirin and gamma globulins in 82.9 and 53.6% of pediatric patients, respectively, after CAA diagnosis. Among the antithrombotic agents, aspirin was used most frequently, followed by dipyridamole (16.9%), heparin (5.8%) and warfarin (4.6). Only 4 patients (0.7%) received clopidogrel. Since most of the pediatric patients (95.7%) were diagnosed with Kawasaki disease, antithrombotic treatments were reasonably used in the clinic. On the basis of the recommendations by the American Heart Association (AHA) guidelines [29], aspirin should be administered at a high dosage (80 to 100 mg/kg/day) with intravenous immunoglobulin (IVIG) in the acute phase because these two medications appear to possess an additive anti-inflammatory effect. However, the duration of high-dose aspirin treatment differs by institution, and most reduce the aspirin dose 48 to 72 h after fever subsides. In certain cases, the duration may be prolonged to 14 days after Kawasaki disease onset.

Aspirin treatment should be adjusted to a low dosage (3 to 5 mg/kg/day) after the acute phase. Platelet aggregation activity remains high during the first 3 months after Kawasaki disease onset and can even last for 1 year after disease onset in some cases. For patients who develop coronary aneurysm due to Kawasaki disease, aspirin may be continued indefinitely to prevent ischemic heart disease and prevent the formation or growth of thrombi by platelet aggregation based on the Japanese Circulation Society (JCS) guidelines [30]. Suggested antiplatelet agents to be used in children with Kawasaki disease include aspirin, flurbiprofen, dipyridamole, ticlopidine and clopidogrel. Choices for anticoagulant drugs include heparin, low-molecular weight heparin (LMWH) and warfarin. In view of the existing CAAs in our patients with Kawasaki disease, antiplatelet treatment for the prevention of cardiac sequela is reasonable.

### Prescribing patterns in adult patients

Regarding medication use in adult patients, we found that antithrombotic agents, particularly aspirin, clopidogrel and heparin, were prescribed more frequently after CAA diagnosis. Cardiovascular medications, oral antidiabetic agents, lipid-lowering agents and nitrates, were also increasingly prescribed after CAA diagnosis. Among the prescriptions, aspirin (75.8%), β-blockers (48.3%), statins (47.6%), metformin (14.4%), sulfonylureas (14.4%) and isosorbide mononitrate (32.9%) were frequently observed in each category. Data regarding prescribing patterns in CAA patients was limited in the previous literature. Most series focused on whether patients received surgery, percutaneous intervention or medical therapy instead of specific drug utilization. Only a few studies reported the prescribing pattern in patients with CAA [31, 32]. Sultana et al. [31] reported that 56, 14 and 70% of patients were prescribed aspirin, warfarin and a combination of aspirin and warfarin, respectively. Other medications, such as calcium channel blockers (CCBs), β-blockers and nitrates, were used in 25, 20 and 34% of patients, respectively. A 97% prescribing rate for aspirin was reported in a series by Almansori et al., [32] in which angiotensin-converting-enzyme (ACE) inhibitors (59%), angiotensin II receptor blockers (ARBs) (34%), CCBs (22%), statins (95%) and nitrates (49%) were also recorded. The great variety of prescribing patterns across studies was mainly affected by coexisting diseases. A previous review by Dahhan [33] suggested that the management of coronary artery ectasia should be based on the underlying etiology. The general medical therapy for patients with coronary artery ectasia includes antiplatelet agents, β-blockers, ACE inhibitor, ARBs, CCBs and statins, especially if another indication is present (e.g., hypertension, dyslipidemia, diabetes mellitus).

Concerning the effect of specific medications, antiplatelets, β-blockers and statins are indicated for accompanying atherosclerotic coronary artery disease. In addition, other drugs have different indications: anticoagulants for secondary prevention of further thrombosis; dihydropyridine CCB for the prevention of vasospasm; and ACE inhibitors for decreasing inflammation.

Our study showed that patients under 20 years of age are exposed to acute respiratory infections, noninfectious gastroenteritis and colitis, allergic rhinitis and contact dermatitis very often. There may be a relationship between Kawasaki disease with allergic diseases and this finding is consistent with previous studies [34, 35]. Kawasaki disease were at an increased risk for allergic diseases compared with the comparison cohort and children with Kawasaki disease had a higher risk of developing atopic dermatitis.

In summary, the management of CAA should be tailored to each individual based on the underlying etiology. Our adult population had a significant burden of cardiovascular disease, which indicated that drugs prescribed for such chronic conditions were reasonable.

### Limitations

To the best of our knowledge, this is the first population-based study investigating the comorbidities and prescribing patterns for CAA patients in Taiwan. We enrolled CAA population in Taiwan, with data obtained from the NHIRD. We are able to provide an explicit age distribution, including pediatric and adult populations, which was different from previous studies that used an angiographic method to enroll adult patients. However, there were some limitations in our study. First, our data were obtained from a claim-based database, and we did not have information on aneurysm features, including the location, morphology, aneurysm size, number of aneurysms and degree of stenosis. In addition, we could not differentiate between CAAs and coronary artery ectasia in this study. Second, we did not have laboratory or image data to determine the severity of artery stenosis, blood pressure values and hemoglobin A1c levels. Some cardiovascular risk factors, such as smoking and body mass index, were also unavailable in the database.

## Conclusion

Both adult and pediatric CAA patients were surveyed in our study. Kawasaki disease was the main cause of CAA in pediatric patients, and aspirin use was increased after CAA diagnosis. Coronary artery disease was the most common comorbidity in adult CAA patients. The most commonly used antithrombic agent after CAA diagnosis in adults was aspirin.

## Data Availability

The data that support the findings of this study are available from NHIRD, but restrictions apply to the availability of these data, which were used under license for the current study and thus are not publicly available. Data are, however, available from the authors upon reasonable request and with permission of the National Health Insurance Research Institutes.

## Abbreviations

ACEIs: Angiotensin-converting enzyme inhibitors
Angio: Coronary angiography
ARBs: Angiotensin II receptor blockers
Cath: Cardiac catheterization
CCBs: Calcium channel blockers
COPD: Chronic obstructive pulmonary disease
CT: Computed tomography
Echo: Echocardiography
MRI: Magnetic resonance imaging
NHIRD: National Health Insurance Research Databases

## References

[1] Syed M, Lesch M (1997). Coronary artery aneurysm: a review. Prog Cardiovasc Dis.

[2] Swaye PS, Fisher LD, Litwin P, Vignola PA, Judkins MP, Kemp HG (1983). Aneurysmal coronary artery disease. Circulation..

[3] Reji R, Nguyen M. Medically managed coronary artery aneurysm without concomitant stenosis. BMJ Case Rep. 2018;2018:bcr–2018–224244. 10.1136/bcr-2018-224244 PMC5961543 29764828

[4] Fang CT, Fang YP, Huang YB, Kuo CC, Chen CY (2017). Epidemiology and risk factors of coronary artery aneurysm in Taiwan: a population based case control study. BMJ Open.

[5] Khouzam RN, Gardner JD, Bomb R, Holden AA (2017). Multi-vessel giant coronary artery aneurysm in an elderly female. Ann Transl Med.

[6] Núñez-Gil IJ, Nombela-Franco L, Bagur R, Bollati M, Cerrato E, Alfonso E (2017). Rationale and design of a multicenter, international and collaborative coronary artery aneurysm registry (CAAR). Clin Cardiol.

[7] B M, Yazici HU, Aydar Y, Ovali C, Nadir A (2016). Role of gender in types and frequency of coronary artery aneurysm and ectasia. Medicine (Baltimore).

[8] Boyer N, Gupta R, Schevchuck A, Hindnavis V, Maliske S, Sheldon M (2014). Coronary artery aneurysms in acute coronary syndrome: case series, review, and proposed management strategy. J Invasive Cardiol.

[9] Lanjewar CP, Sharma A, Sheth T (2009). IVUS-guided management of late stent malaposition with peri-stent restenosis with coronary artery aneurysm following drug-eluting stent implantation (paxlitaxel-eluting stent). J Invasive Cardiol.

[10] Alfonso F, Pérez-Vizcayno MJ, Ruiz M, Suárez A, Cazares M, Hernández R (2009). Coronary aneurysms after drug-eluting stent implantation: clinical, angiographic, and intravascular ultrasound findings. J Am Coll Cardiol.

[11] Tunick PA, Slater J, Kronzon I, Glassman E (1990). Discrete atherosclerotic coronary artery aneurysms: a study of 20 patients. J Am Coll Cardiol.

[12] Bhindi R, Testa L, Ormerod OJ, Banning AP (2009). Rapidly evolving giant coronary aneurysm. J Am Coll Cardiol.

[13] Chia HM, Tan KH, Jackson G (1997). Non-atherosclerotic coronary artery aneurysms: two case reports. Heart.

[14] Dajani AS, Taubert KA, Gerber MA, Shulman ST, Ferrieri P, Freed M (1993). Diagnosis and therapy of Kawasaki disease in children. Circulation.

[15] Senzaki H (2006). The pathophysiology of coronary artery aneurysms in Kawasaki disease: role of matrix metalloproteinases. Arch Dis Child.

[16] Newburger JW, Takahashi M, Burns JC, Beiser AS, Chung KJ, Duffy CE (1986). The treatment of Kawasaki syndrome with intravenous globulin. N Engl J Med.

[17] Gelb BD (2006). Marfan’s syndrome and related disorders--more tightly connected than we thought. N Engl J Med.

[18] Cohen P, O'Gara PT (2008). Coronary artery aneurysms: a review of the natural history, pathophysiology, and management. Cardiol Rev.

[19] Friedman K, Gauvreau K, Hamaoka-Okamoto A, Tang A, Berry E, Tremoulet A (2016). Coronary artery aneurysms in Kawasaki disease: risk factors for progressive disease and adverse cardiac events in the US population. J Am Heart Assoc.

[20] Pahlavan PS, Niroomand F (2006). Coronary artery aneurysm: a review. Clin Cardiol.

[21] Demopoulos VP, Olympios CD, Fakiolas CN, Pissimissis EG, Economides NM, Adamopoulou E (1997). The natural history of aneurysmal coronary artery disease. Heart.

[22] Nichols L, Lagana S, Parwani A (2008). Coronary artery aneurysm: a review and hypothesis regarding etiology. Arch Pathol Lab Med.

[23] Chang LY, Chang IS, Lu CY, Chiang BL, Lee CY, Chen PJ (2004). Epidemiologic features of Kawasaki disease in Taiwan, 1996-2002. Pediatrics..

[24] Huang WC, Huang LM, Chang IS, Chang LY, Chiang BL, Chen PJ (2009). Epidemiologic features of Kawasaki disease in Taiwan, 2003-2006. Pediatrics..

[25] Lin MC, Lai MS, Jan SL, Fu YC (2015). Epidemiologic features of Kawasaki disease in acute stages in Taiwan, 1997-2010: effect of different case definitions in claims data analysis. J Chin Med Assoc.

[26] Boles U, Eriksson P, Zhao Y, Henein MY (2010). Coronary artery ectasia: remains a clinical dilemma. Coron Artery Dis.

[27] Pinar Bermúdez E, López Palop R, Lozano Martínez-Luengas I, Cortés Sánchez R, Carrillo Sáez P, Rodriguez Carreras R (2003). Coronary ectasia: prevalence, and clinical and angiographic characteristics. Rev Esp Cardiol.

[28] Valente S, Lazzeri C, Giglioli C, Sani F, Romano SM, Margheri M (2007). Clinical expression of coronary artery ectasia. J Cardiovasc Med (Hagerstown).

[29] Newburger Jane W., Takahashi Masato, Gerber Michael A., Gewitz Michael H., Tani Lloyd Y., Burns Jane C., Shulman Stanford T., Bolger Ann F., Ferrieri Patricia, Baltimore Robert S., Wilson Walter R., Baddour Larry M., Levison Matthew E., Pallasch Thomas J., Falace Donald A., Taubert Kathryn A. (2004). Diagnosis, Treatment, and Long-Term Management of Kawasaki Disease. Circulation.

[30] JCS Joint Working Group. Guidelines for diagnosis and management of cardiovascular sequelae in Kawasaki disease (JCS 2013). Circ J. 2014;78(10):2521–62. 10.1253/circj.cj-66-0096 25241888

[31] Sultana R, Sultana N, Ishaq M, Samad A (2011). The prevalence and clinical profile of angiographic coronary ectasia. J Pak Med Assoc.

[32] Almansori MA, Elsayed HA (2015). Coronary artery ectasia - a sample from Saudi Arabia. J Saudi Heart Assoc.

[33] Dahhan A (2015). Coronary artery ectasia in atherosclerotic coronary artery disease, inflammatory disorders, and sickle cell disease. Cardiovasc Ther.

[34] Kuo H-C, Chang W-C, Yang KD, Yu H-R, Wang C-L, Ho S-C, Yang C-Y (2013). Kawasaki disease and subsequent risk of allergic diseases: a population-based matched cohort study. BMC Pediatr.

[35] Woon PY, Chang WC, Liang C-C, Hsu CH, Klahan S, Huang Y-H, Chang W-P, Kuo H-C (2013). Increased risk of atopic dermatitis in preschool children with Kawasaki disease: a population-based study in Taiwan. Evid Based Complement Alternat Med.

